# Carbon Nanohorns and Their Nanohybrid/Nanocomposites as Sensing Layers for Humidity Sensors—A Review

**DOI:** 10.3390/polym17162198

**Published:** 2025-08-12

**Authors:** Bogdan-Catalin Serban, Octavian Buiu, Marius Bumbac, Niculae Dumbrăvescu, Mihai Brezeanu, Ursăchescu Matei-Gabriel, Vlad Diaconescu, Maria Ruxandra Sălăgean, Cornel Cobianu

**Affiliations:** 1 National Institute for Research and Development in Microtechnologies, IMT-Bucharest, Str. Erou Iancu Nicolae 126A, 077190 Voluntari, Romania; octavian.buiu@imt.ro (O.B.); nicolae.dumbravescu@imt.ro (N.D.); 2 Sciences and Advanced Technologies Department, Faculty of Sciences and Arts, Valahia University of Târgoviște, Aleea Sinaia 13, 130004 Târgoviște, Romania; 3 Faculty of Electronics, Telecommunications, and IT, National University of Science and Technology Politehnica Bucharest, Bd Iuliu Maniu 1-3, 061071 Bucharest, Romania; mbrezeanu@upb.ro; 4 National College “B. P. Hasdeu”, Bulevardul Gării, 1, 120018 Buzău, Romania; ursachescumatei@gmail.com; 5 Faculty of Medicine, University of Medicine and Pharmacy “Carol Davila”, Dionisie Lupu Street, No. 37, Sector 2, 030167 Bucharest, Romania; diaconescuvld506@gmail.com; 6 National College “Saint Sava”, 23 General H. M. Berthelot Street, 010164 Bucharest, Romania; salagean.ruxandra@gmail.com; 7 Academy of Romanian Scientists (ARS), Str. Ilfov Nr. 3, Sector 5, 050044 Bucharest, Romania; cornel.cobianu@imt.ro; 8 eBio-hub Center of Excellence in Bioengineering, National University of Science and Technology Politehnica Bucharest, 061103 Bucharest, Romania

**Keywords:** carbon nanohorns, resistive sensors, hydrophilic polymers, nanocomposites, monohybrids

## Abstract

Carbon nanohorns (CNHs), along with their nanocomposites and nanohybrids, have shown significant potential for humidity (RH) monitoring at room temperature (RT) due to their exceptional physicochemical and electronic properties, such as high surface area, tunable porosity, and stability in nanocomposites. Resistive sensors incorporating CNHs have demonstrated superior sensitivity compared to traditional carbon nanomaterials, such as carbon nanotubes and graphene derivatives, particularly in specific RH ranges. This review highlights recent advancements in CNH-based resistive RH sensors, discussing effective synthesis methods (e.g., arc discharge and laser ablation) and functionalization strategies, such as the incorporation of hydrophilic polymers or inorganic fillers like graphene oxide (GO) and metal oxides, which enhance sensitivity and stability. The inclusion of fillers, guided by Pearson’s Hard–Soft Acid–Base (HSAB) theory, enables tuning of CNH-based sensing layers for optimal interaction with water molecules. CNH-based nanocomposites exhibit competitive response and recovery times, making them strong candidates for commercial sensor applications. However, challenges remain, such as optimizing materials for operation across the full 0–100% RH range. This review concludes with proposed research directions to further enhance the adoption and utility of CNHs in sensing applications.

## 1. Introduction

**Figure 1:**
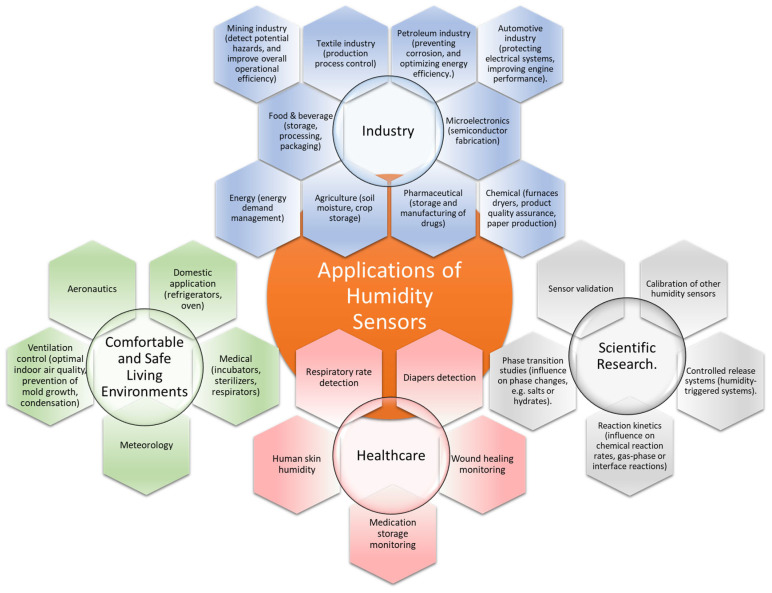
Several application areas for humidity sensors.

Relative humidity (RH), a key environmental parameter that significantly impacts all life forms, is defined as the ratio of the amount of water vapor in the air to the amount of water vapor the air can hold at a specific temperature [1]. The RH level affects human ability to regulate body temperature, influences the growth of harmful microorganisms, and can impact the quality of indoor and outdoor environments. Exposure to high RH levels can lead to discomfort, trigger respiratory issues such as bronchoconstriction, and increase the risk of heat-related illnesses. On the contrary, low RH can promote dryness of the skin, a dry throat, irritated eyes, and constricted respiratory passages [2,3,4]. Monitoring RH is crucial in various commercial, industrial, and residential applications, some of which are depicted in Figure 1 [5,6,7,8].

Given the above, numerous principles and technologies have been developed over time for performing RH monitoring. The need for accurate and reliable RH measurements has driven innovation in sensor technology, resulting in improvements in range response, linearity, response time, drift, cost-effectiveness, accuracy, and other key metrics [9]. Among the types of sensors used so far in RH monitoring, such as capacitive [10], thermal conductivity [11], magnetoelastic [12], electrochemical [13], surface acoustic wave [14], bulk acoustic wave [15], piezoelectric/triboelectric nanogenerators [16], ion gradient [17] and optical [18], resistive sensors are an attractive option [19]. Simple design, small size, low cost, robustness, quick response times, and wide measurement ranges are just some of the advantages that resistive sensors offer in RH monitoring.

The nature of the sensing layer, a key element of a resistive RH sensor, plays a crucial role in manufacturing a resistive device with optimal performance. Consequently, a wide range of materials have been tested as a sensing layer within the design of resistive RH sensors: conducting polymers, such as polypyrrole [20], dielectric polymers, such as polyimide [21], semiconductors, such as cadmium sulfide [22], ZnO [23], SnO_2_ [24], polyelectrolytes [25], perovskites [26], nanoclays like attapulgite [27], sepiolite [28], halloysite [29], and so forth.

Carbonic materials have also been widely reported as sensing films in the manufacture of RH sensors. Over the last few decades, these materials have gained popularity due to their outstanding properties, including high chemical and thermal stability, versatile covalent functionalization to optimize the surface for proper interaction with water molecules, fast charge transfer, a large surface area, low cost, and facile and scalable synthesis [30]. Several carbon-based materials and their nanohybrids/nanocomposites used as sensing elements in the design of RH-resistive sensors are listed in Table 1.

In recent years, carbon nanohorns (CNHs) and their nanocomposites or nanohybrids have shown promise as materials for gas-sensing applications, owing to their unique properties and potential to enhance gas detection performance, as suggested by a limited but growing body of research [56,57].

This review article focuses on the latest advancements and new perspectives of CNHs (pristine and functionalized), including nanocomposites and nanohybrids, as sensing materials for RH monitoring using resistive sensors. The review is organized into six main parts. In the first part, the main approaches to synthesizing CNHs are described. Significant attention will be devoted to the functionalization of these nanocarbonic structures to optimize their sensing properties towards water molecules. The second part primarily focuses on the most relevant physicochemical and electronic properties of CNHs, which make them valuable sensing materials for RH monitoring. The third part briefly presents the design of the RH-resistive sensors, which employ CNH-based materials as sensing films. The fourth part includes the synthesis and performance of several CNH-based sensors for RH monitoring within the design of resistive sensors. Thus, pristine and functionalized CNHs, nanocomposites with several hydrophilic polymers, and nanohybrids with several semiconducting metal oxides are analyzed and compared in terms of sensitivity, response time, and recovery time. The fifth part of this review presents several sensing mechanisms involved in RH detection, as well as an analysis of how the properties of each component in the nanocomposite/nanohybrid and the mutual interaction between them influence the discussed RH-sensing mechanisms. Finally, in the sixth part of this review, possible opportunities and future research directions are presented. Ultimately, this review seeks to address the question: is the production of commercial sensors based on CNHs feasible?

## 2. Structure and Synthesis of Pristine CNHs and Their Derivatives Used in Resistive RH Monitoring

### 2.1. Structure of CNHs

**Figure 2:**
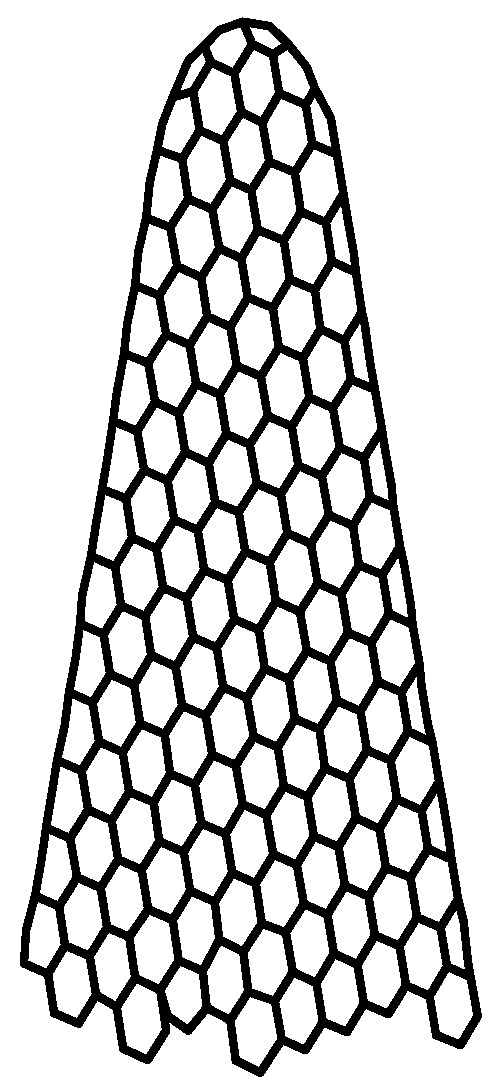
The structure of CNHs.

CNHs (Figure 2) are conical carbon nanocages with cone angles of about 20°, constructed from a sp^2^ carbon sheet with an average length of 40 to 50 nm and a diameter range of 2–5 nm [58]. In contrast to carbon nanotubes (CNTs), which are essentially rolled-up graphene sheets, CNHs have a more complex structure that includes a mix of pentagons, hexagons, and heptagons. This structural diversity contributes to their unique chemical properties and potential applications [59].

CNHs tend to aggregate into three distinct structural arrangements: “dahlia-like”, “bud-like”, and “seed-like”. These structures differ in their overall morphology and the arrangement of the individual horn-shaped nanohorns within the aggregate: “dahlia-like” resembles a dahlia flower with many petals; “bud-like” resembles a flower bud; and “seed-like” is a more compact arrangement. For many years, this structural characteristic was considered a significant drawback in the functionalization of individual CNHs. However, this limitation has recently been overcome by using a novel technique, as recently reported, to separate these supramolecular architectures into individual CNHs [60].

### 2.2. Synthesis of Pristine CNHs

In recent decades, several approaches to synthesizing pristine CNHs have been developed. All these methods involve the injection of energy to vaporize and rearrange a graphite target, followed by rapid quenching, typically in an inert gas atmosphere. The morphology, size, and purity of CNHs are modulated through variations in various operational parameters, including temperature, current, voltage, and pressure, among others [61].

One of the most commonly used CNH synthesis routes is that of arc discharge, which involves passing a high current between two graphite electrodes in atmospheric air. The purity of synthesized CNHs is higher than 90% [62]. The arc discharge approach comprises three steps: carbon vaporization, recondensation (where the vaporized carbon atoms recondense as they cool, forming CNHs and other carbon nanomaterials), and purification. The surrounding atmosphere (e.g., air, Ar, CO_2_, or CO), gas flow rate, and arc current play a crucial role in determining the type, size, and morphology of the synthesized CNHs [63]. H. Wang et al. [64] reported a cost-effective synthesis of a mixture of “dahlia-like” and “bud-like” CNHs based on arc discharge between two graphite rods submerged in liquid nitrogen. 

The synthesis of CNHs by CO_2_ laser ablation of graphite in the absence of any catalyst was also performed in recent decades [65]. Synthesis via Joule heating [66] and through inductively coupled plasma [67] represent two other feasible methods for the mass production of CNHs. It is essential to note that the synthesis of CNHs is conducted in the absence of a catalyst, which is a significant advantage compared to, for example, the synthesis of CNTs [60].

### 2.3. Synthesis of Functionalized CNHs for Resistive RH Monitoring

Due to their predominantly nonpolar carbon structure, pristine CNHs are generally hydrophobic. Therefore, to increase their affinity toward water molecules, their surface must be functionalized to make them more hydrophilic. Oxidation of CNHs with concentrated nitric acid, H_2_O_2_/hv, or H_2_O_2_/H_2_SO_4_, can introduce carboxyl groups onto the surface of nanocarbonic materials, enhancing their hydrophilicity and dispersibility in polar solvents such as water, isopropanol, and ethanol [68]. Treatments like plasma exposure or oxidation can introduce polar functional groups (like carboxyl or hydroxyl groups) onto the CNH surface.

**Figure 3:**
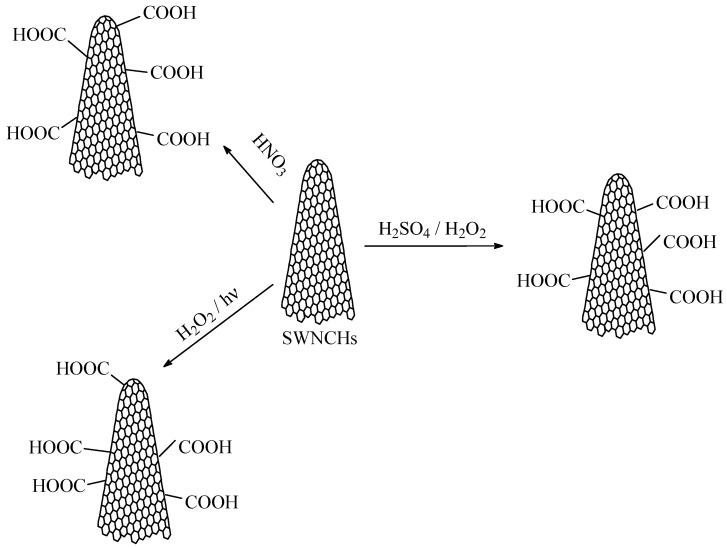
The synthesis of oxidized carbon nanohons (CNHox).

The synthesis of oxidized CNHs (CNHox) is carried out using oxygen plasma treatment and water plasma treatment, as depicted in Figure 3. Both types of hydrophilization allow the functionalization of the CNHs by grafting carboxyl, hydroxyl, carbonyl, and epoxy groups. The degree of hydrophilization of the CNHs necessary to achieve superior RH-sensing performance (high sensitivity, low response time, low hysteresis, etc.) can be modulated by adjusting the plasma power and exposure time [69].

**Figure 4:**
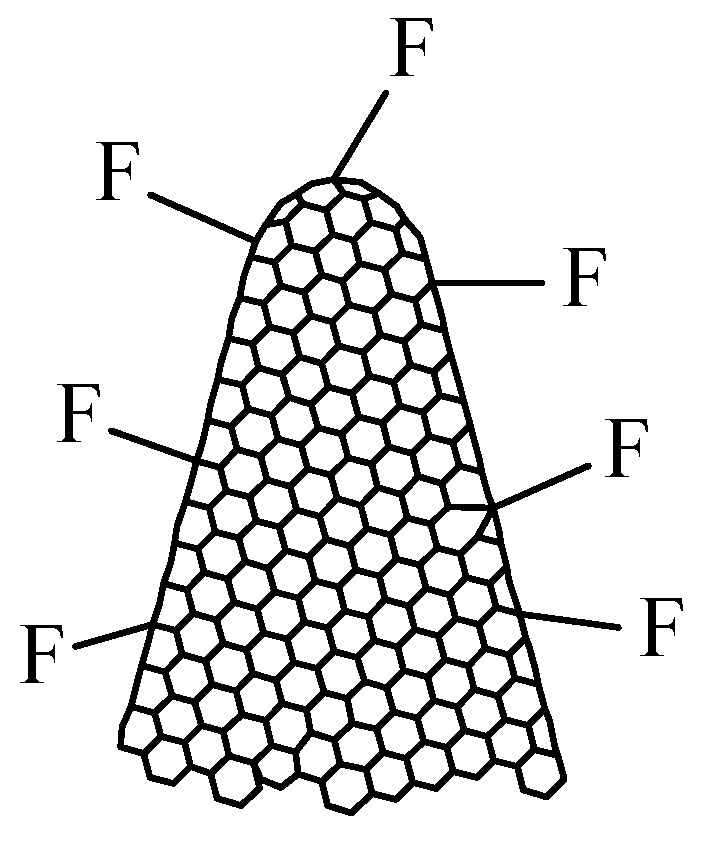
The structure of fluorinated carbon nanohorns (CNHs-F).

Fluorinated carbon nanohorns (CNHs-F), with the structure presented in Figure 4, can also be synthesized and represent a viable option for resistive RH monitoring [70]. The synthesis of CNHs-F is performed by plasma treatment of CNHs in F_2_ and N_2_ (volume mixture 1:10) at a pressure of 0.5 bar in a nickel reactor at RT. The injection time is 4 min, and the exposure time ranges from 2 to 8 min.

**Figure 5:**
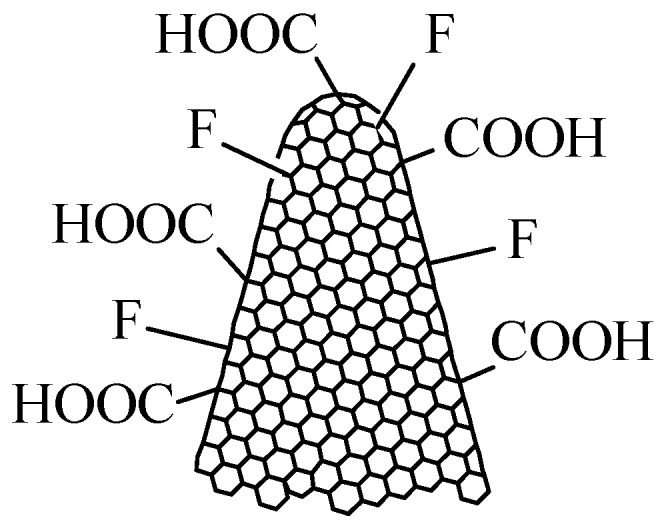
The structure of oxyfluorinated carbon nanohorns (CNHox-F).

Another functionalized carbon nanohorn–based material, oxyfluorinated carbon nanohorns (CNHox-F, with the structure inserted in Figure 5), can be synthesized in a two-step plasma procedure [71]. The synthesis of CNHox-F begins by treating CNHs in a volumetric mixture of Ar-O_2_ (3:1) in a quartz tube at a pressure of 3 Torr and room temperature (RT). The injection time is 5 min, and the exposure time ranges from 2 to 4 min. The fluorination of CNHox is carried out by treating ox-CNHs in a F_2_ and N_2_ plasma (volumetric mixture 1:10) at a pressure of 0.5 bar in a nickel reactor at RT. The injection time is 4 min, and the exposure time ranges from 2 to 4 min.

The use of CNHox-F as a sensitive layer has several significant advantages. Firstly, the presence of oxygenated functions, generated by treating simple nanocarbon materials with Ar-O_2_ plasma, ensures the degree of hydrophilicity necessary for interaction with water. Secondly, due to their increased electronegativity, fluorine atoms enhance the polarity of the nanocarbon material’s surface, creating temporary dipoles that facilitate interaction with water molecules.

## 3. Properties of CNHs

Due to their unique nanostructure, CNHs exhibit outstanding properties as follows:

- *High thermal conductivity*—The thermal conductivity of CNHs is about 6250 W/m K, larger than that of other nanocarbonic materials, such as CNTs [72,73]
- *High surface area*—CNHs have a large specific surface area, which is a key feature for applications such as catalysis and adsorption [74]
- *Excellent porosity*—The partial oxidation of CNHs gives direct access to internal pores via the generation of nanowindows onto the skeleton of CNHs. Holes can be easily created in pristine CNHs by heat treatment under oxidative and/or acidic conditions [75]
- *High adsorption capacity*—[76]
- *Thermal stability*—CNHs generally exhibit good thermal stability, particularly in inert atmospheres. In air, the oxidation of SWNHs starts above 300 °C and is completed at 720 °C. CNHs can remain stable in a vacuum up to 1800 °C [77]
- *High purity*—CNHs can be synthesized with high purity, often exceeding 95%, and without the need for metal catalysts [78]
- *Chemical stability*—CNHs generally exhibit good corrosion resistance, particularly when compared to some other nanocarbonic materials [79]
- *Low toxicity*—Several experiments conducted in recent years show that CNHs have low toxicity [80]
- *Catalytic properties*—CNHs can act both as catalysts and catalyst supports for metal nanoparticles [81]
- *Good electrical conductivity*—CNHs generally exhibit lower electrical conductivity compared to CNTs; however, the conductivity of both materials can be influenced by several parameters, including purity, structure, and type of synthesis. The electrical percolation threshold of carbon nanohorns and their derivatives in several hydrophilic polymers is a key parameter in the evaluation of resistive sensing capabilities of nanocomposites based on CNHs for different gases and RH [82,83]
- *Facile covalent and noncovalent functionalization*—[84]

We anticipate that all these features will position CNHs as an appropriate substitute for CNTs in the near future.

## 4. Structure of CNH-Based Resistive RH Sensors

**Figure 6:**
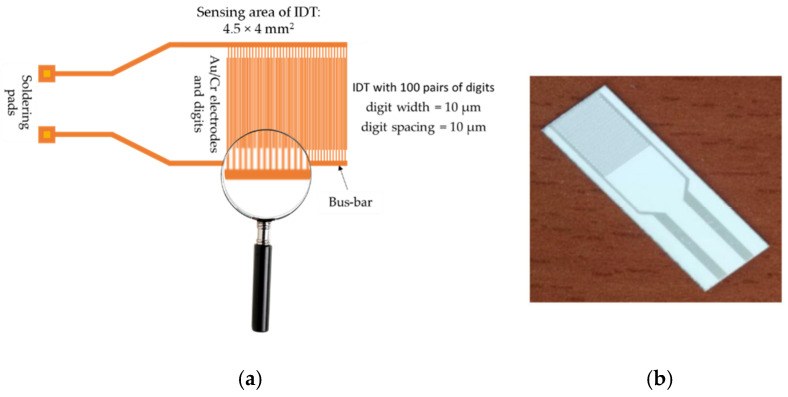
(**a**) The metal stripes of IDT (interdigitated structure), and (**b**) the polyimide-based sensing structure used for resistive RH monitoring. Alternatively, a flexible substrate made from polyimide (3.95 × 3.95 mm^2^), with gold interdigitated electrodes (Au IDTs), as depicted in (**b**), can also be used for a CNH-based resistive RH sensor [82].

CNH-based resistive RH sensors typically include a substrate, a sensing layer, and two metal electrodes. The most used sensing structure (presented in Figure 6a) is manufactured on a Si substrate (470 µm thick), covered by a SiO_2_ layer (1 µm thick). The metal stripes of interdigitated transducer (IDT) electrodes typically consist of Cr (10 nm thickness) and Au (100 nm thickness). The width of the electrodes is about 200 µm, with a separation of 6 mm between them. The digits of the electrodes have a width and spacing of 10 µm.

At the same time, in a simple experimental approach, Selvam et al. [85] have demonstrated the use of cellulose sheet as a flexible substrate. The increase in resistance with the RH level was measured between two nickel electrodes, placed 5 mm apart.

## 5. RH-Resistive Sensors Based on CNHS and Their Nanocomposites/Nanohybrids

The idea of using CNHs and their derivative as a sensing layer within the design of a resistive RH sensor was recently introduced [86]. Oxidized carbon nanohorns (CNHox)—carboxymethylcellulose and CNHox-agarose—are the first two carbon nanohorn–based nanocomposites proposed to be used as a sensitive film for resistive monitoring of RH.

### 5.1. Oxidized CNHs as Sensing Layers in RH-Resistive Sensors

**Figure 7:**
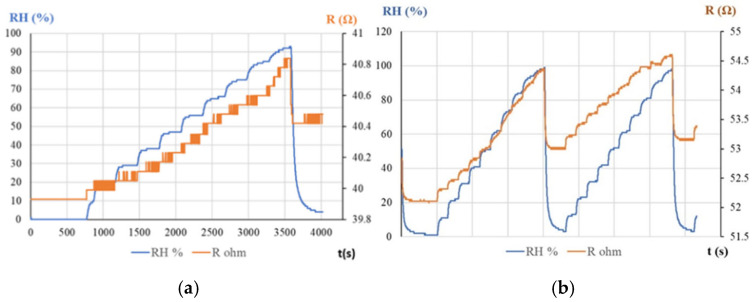
The RH response of the CNHox-based sensor in: (**a**) humid nitrogen (red curve) vs. the RH response of the Sensirion RH sensor (blue curve); and (**b**) humid air (red curve) vs. the RH response of the Sensirion RH sensor (blue curve) [87].

Serban et al. used, for the first time, a sensitive layer based exclusively on a derivative of CNHs, namely, CNHox [87,88]. An essential feature of these sensors was the use of a CNH concentration higher than the percolation threshold in the polymeric matrix, thereby providing lower resistance values. The resistive response of the RH sensor used was investigated by applying a current between the two Cr and Au electrodes deposited on a Si/SiO_2_ substrate and measuring the resistance when varying the RH from 0% up to 90%, both in a humid nitrogen environment (Figure 7a) and in humid air (Figure 7b). The resistance of the CNHox-based sensing film increased when the RH increased. The sensor response was compared to that of a commercial sensor (Sensirion^®^ RH sensor, Sensirion AG, Stäfa, Switzerland). 

**Figure 8:**
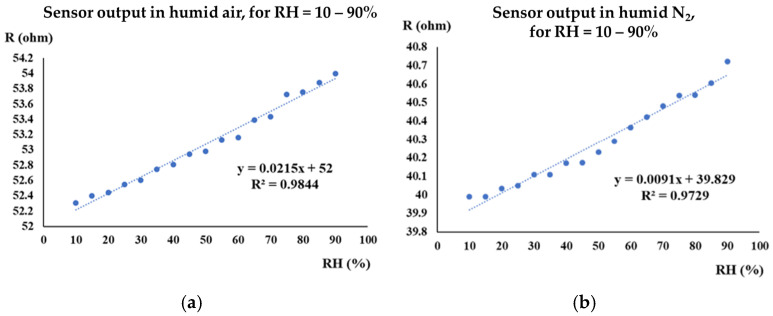
The transfer function of the CNHox-based sensor in: (**a**) humid air (RH = 10–90%); and (**b**) humid nitrogen (RH = 10–90%) [87].

The manufactured CNHox-based sensor exhibited good linearity in both humid air (R^2^ = 0.9844, Figure 8a) and humid nitrogen (R^2^ = 0.9729, Figure 8b).

The sensitivity was approximately 2 times lower in humid nitrogen compared to humid air (9.1 mΩ/RH unit compared to 21 mΩ/RH unit). The response time of the CNHox-based RH sensor in humid nitrogen was 8 s, while in humid air it was 3 s.

### 5.2. Nanocomposite-Based CNHs as Sensing Layers in RH-Resistive Sensors

#### 5.2.1. Pristine CNHs–Hydroxyethylcellulose as Sensing Layer in RH-Resistive Layers

By combining the appropriate electrical and mechanical properties of pristine CNHs and the hydrophilic properties of hydroxyethylcellulose, Selvam et al. [85] synthesized a nanocomposite with different loading concentrations of CNHs (5–50 wt%). The resistance of the nanocomposite was shown to increase with the RH. The response time of the CNHs/cellulose-based sheet was 4 s, while the recovery time was 13 s.

#### 5.2.2. CNHox–PVP as Sensing Layer in RH-Resistive Layers

**Figure 9:**
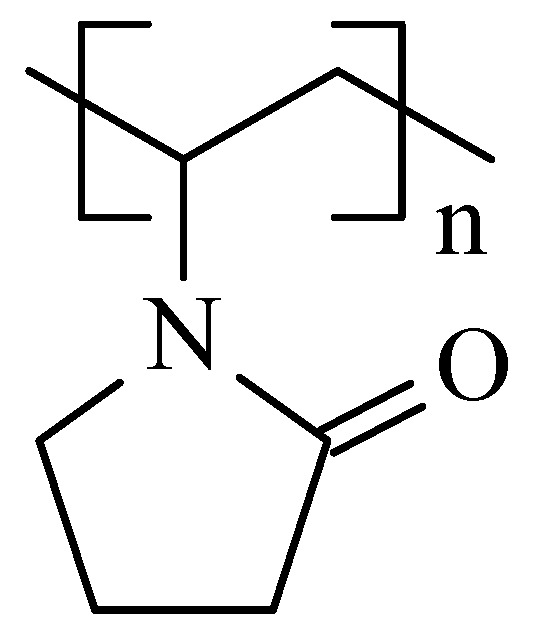
The structure of PVP.

**Figure 10:**
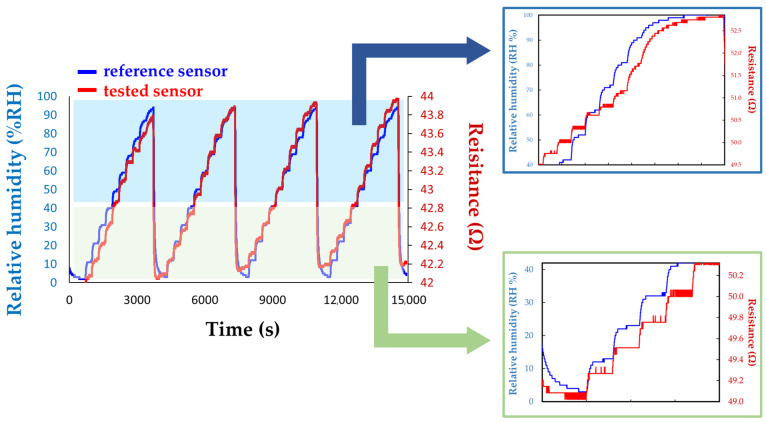
Comparison between the response of the Sensirion commercially available RH sensor (blue line) and the manufactured CNHox-PVP-based (1/2 *w*/*w* ratio) RH sensor (red line).

To further improve the sensitivity to water molecules, Serban et al. combined a hydrophilic type of CNH, namely, CNHox, with a hydrophilic polymer, namely, PVP (the structure is depicted in Figure 9), at 1/1 and 2/1 *w*/*w* ratios [89], significantly above the percolation threshold of CNHox in PVP. The synthesis of the nanocomposite was shown to be very simple. Initially, both the nanocarbonic material and the PVP were dispersed in deionized water. The CNHox-PVP-deionized water dispersion was further deposited by the drop-casting technique on the IDT structure previously deposited on the Si/SiO_2_ substrate to generate the sensing film, in which CNHox and PVP were in a 1/2 *w*/*w*/ratio. The sensing layer was then heated at 80 °C for one hour in a vacuum. The sensing capabilities of the manufactured RH detector employing the novel sensing film were explored in a humid nitrogen atmosphere for different RH values and compared with the response of a Sensirion commercial RH sensor (Figure 10). The sensor exhibits a quasi-similar response to that of a commercially available capacitive RH sensor. Experimental data reveal a linear relationship between R and RH for RH < 40%, and a second-order polynomial function variation for RH > 40% in a humid nitrogen atmosphere. The response time of the proposed sensor structure was in the 5.5–5.9 s range.

#### 5.2.3. CNHox—Poly(ethylene glycol)-blockpoly(propylene glycol)-block-poly(ethylene glycol) (PEG–PPG–PEG) as Sensing Layer in RH-Resistive Layers

**Figure 11:**
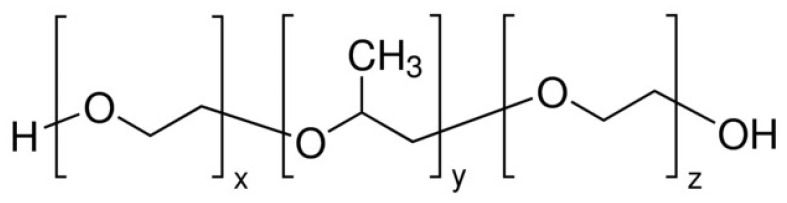
The structure of PEG–PPG–PEG.

**Figure 12:**
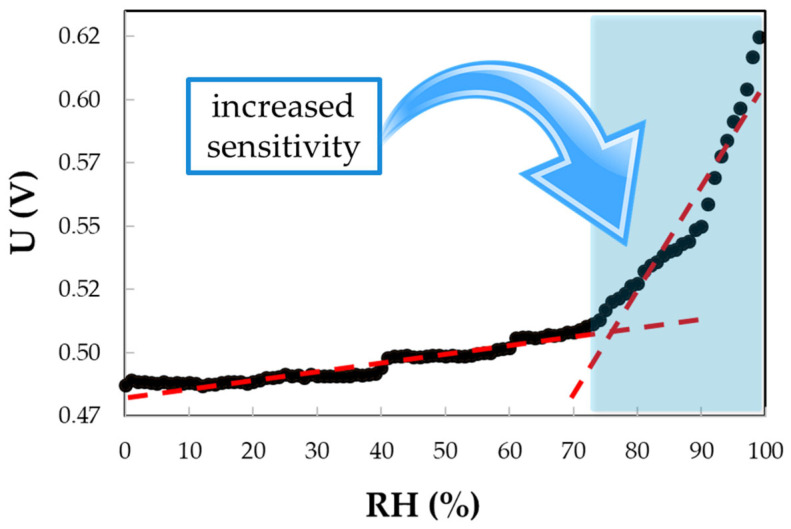
The output signal (voltage) measured when a constant current (0.1 A) is applied to the IDT RH-sensing structure, employing the PEG–PPG–PEG nanocomposite as the sensing layer, for variations in RH from 0% to 98%.

A matrix nanocomposite based on CNHox and PEG–PPG–PEG (the structure of which is depicted in Figure 11) was reported as a sensing layer in the resistive monitoring of RH [89]. In the synthesis process, CNHox and PEG–PPG–PEG (1/6 *w*/*w* ratio) were dispersed in deionized water, subjected to magnetic stirring for three hours at RT, and spin-coated on a Si/SiO_2_ substrate. The RH detection experiments were conducted by applying a current between the two electrodes: Cr with a 10 nm thickness and Au with a 100 nm thickness. The electrode width was approximately 200 mm, with a separation of 6 mm between them. The voltage difference was measured as the RH varied from 0% to 98% (a constant current of 0.1 A was passed through the sensing device). As presented in Figure 12, for RH < 60%, the voltage had a relatively low increase with RH, while for RH > 70%, the sensing device exhibited a stronger RH sensitivity. For the entire RH domain, the electrical resistance of the sensing film increases with RH.

#### 5.2.4. GO-CNHox–PVP as Sensing Layer in RH-Resistive Layers

**Figure 13:**
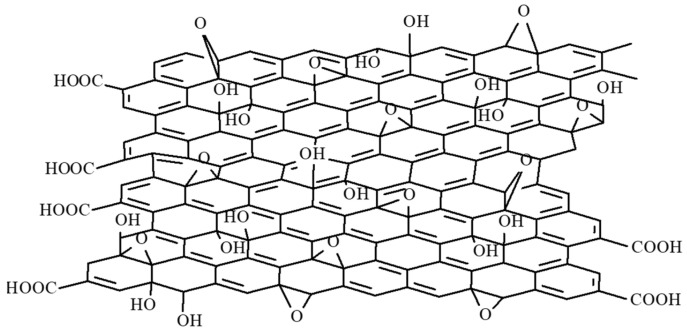
The structure of GO.

A matrix nanocomposite based on GO (the structure is depicted in Figure 13), CNHox, and PVP at mass ratios of 1/1/1, 1/2/1, and 1/3/1 *w*/*w*/*w* was recently reported as the sensing layer within the design of RH-resistive sensors [90,91]. PVP is a well-known binder, while GO disperses CNHox, increasing the specific surface area of the RH-sensitive layer [90]. The synthesis of the ternary nanocomposite was conducted in an ultrasonic bath using isopropanol. At the end of the synthesis process, annealing of the sensing film was achieved using a two-step procedure: a thermal treatment at 353 K and 2 mbar for 20 h, followed by a thermal treatment at 383 K and 2 mbar for 90 h.

**Figure 14:**
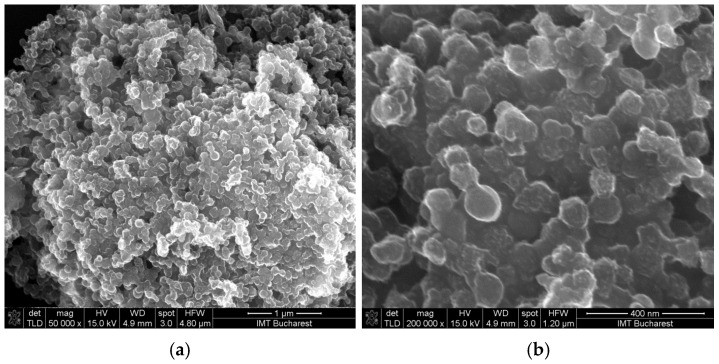
SEM of the GO–CNHox–PVP–based sensing layer at 1:1:1 *w*/*w*/*w* ratio: (**a**) 50,000× magnification; (**b**) 200,000× magnification.

**Figure 15:**
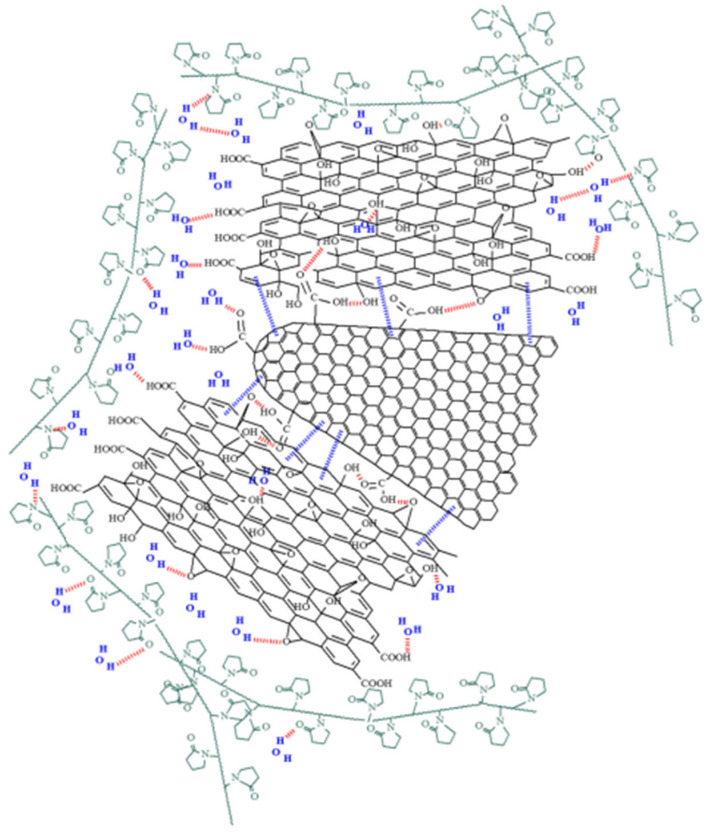
Mutual interactions for the supermolecule generated from CNHox, GO, and PVP [90].

Scanning electron micrographs (SEM) of the GO–CNHox–PVP RH-sensing film deposited onto the Si/SiO_2_ substrate reveal a homogeneous surface, as depicted in Figure 14. Multiple mutual interactions between CNHox, GO, and PVP (as shown in Figure 15), including hydrogen bonds, hydrophobic interactions, and π-π interactions, form a supramolecular aggregate that is a key element in the RH monitoring process.

**Figure 16:**
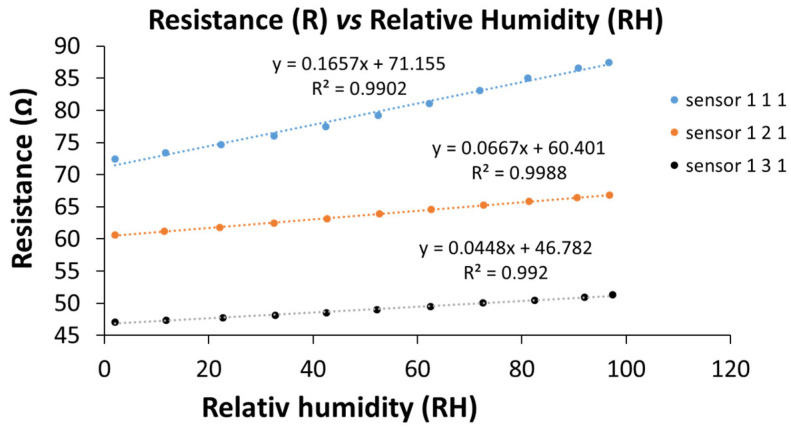
The transfer function of the GO–CNHox–PVP-based (at 1/1/1, 1/2/1, and 1/3/1 *w*/*w*/*w* mass ratios) RH sensors in humid nitrogen (RH = 0–100%) [90].

The linearity of the RH response of the manufactured resistive sensors (sensor 111, sensor 121, and sensor 131 stand for GO–CNHox–PVP at the corresponding 1:1:1, 1:2:1, and 1:3:1 *w*/*w*/*w* mass ratios, respectively), tested in humid nitrogen (for the whole RH range), was shown to be excellent, as depicted in Figure 16.

**Figure 17:**
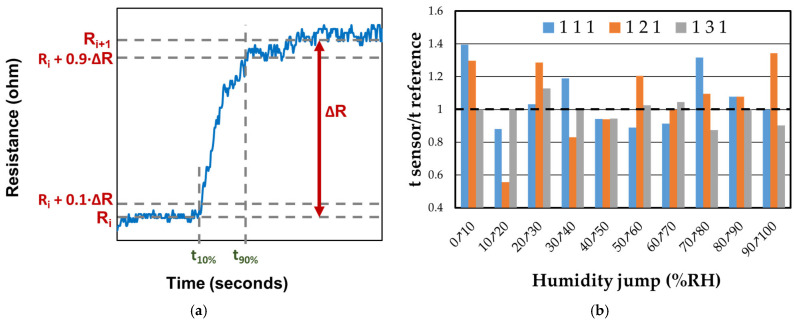
(**a**) The procedure of calculating the response time as the difference between t_90%_ and t_10%_; and (**b**) the ratio of response times of RH sensors 111, 121, and 131 humidity sensors at RT relative to the response time of the reference sensor.

The response and recovery times of sensors 111, 121, and 131 were calculated as the difference between t_90%_ and t_10%_, as shown in Figure 17a. The response time was measured during an increase in RH by 10%, while the recovery time was measured during a decrease in RH from 100% to 0%. It was observed that all sensors exhibited better response times compared to the reference sensor in most situations. In Figure 17b, it can be seen that sensor 131 exhibits consistent behavior in terms of response time, regardless of the humidity change. This sensor contains the highest proportion of CNHOx (1/3/1 = graphene oxide/oxidized carbon nanohorns/PVP), which could be associated with a more dynamic response of CNHOx when water molecules interact with the sensing structure.

The manufactured sensors 111, 121, and 131 had a shorter recovery time compared to the commercial RH sensor when the relative humidity values were decreased from 100% RH to 0% (62 vs. 121, 73 vs. 111, 73 vs. 114).

#### 5.2.5. Pristine CNHs-PVP as Sensing Layer in RH-Resistive Layers

To optimize the hydrophobic-hydrophilic ratio of CNH-based sensing layers used in resistive RH sensors, Serban et al. developed a sensing layer based on CNH-PVP at a 9:1 *w*/*w* ratio [92,93]. Considering the low solubility of CNHs in water and their hydrophobicity, the nanocomposite was synthesized in dimethylformamide (DMF) using an ultrasonic bath. The CNHs-PVP-DMF dispersion was deposited on a polyimide substrate employing gold electrodes. The CNH-PVP sensing film was annealed for two hours at 100 °C under a vacuum.

**Figure 18:**
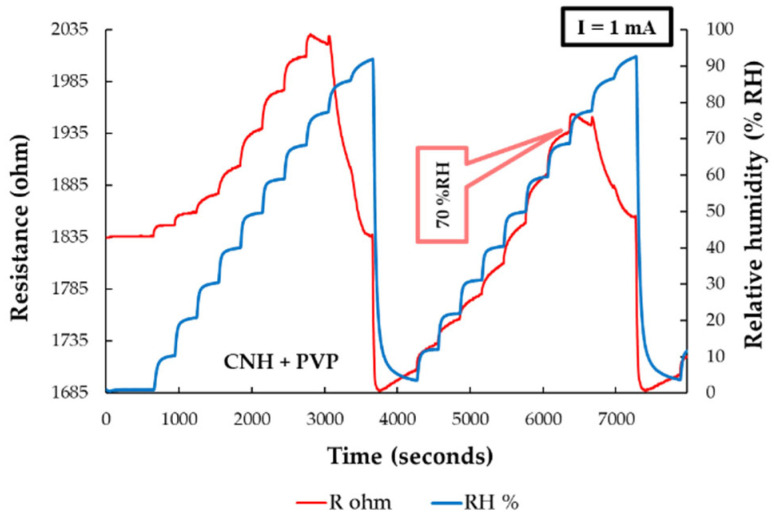
Resistance versus RH variation for the manufactured CNH–PVP–based sensor (red curve) and the reference commercial sensor (blue curve) [93].

The RH detection experimental measurements of the sensor using the CNH-PVP nanocomposite as the sensing film were conducted by applying a current (0.5–1 A) and measuring the voltage difference between the electrodes over the entire RH range (0% to 100%). The response of a CNH–PVP–based manufactured RH sensor was compared to that of a commercial sensor. The resistance of the CNH–PVP–based sensing layer increased when varying RH from 0% to 70%. Once the 70% RH value was reached, the resistance began to decrease with increasing RH. For RH larger than 90%, the resistance started to rise again, as depicted in Figure 18. The combination of different sensing mechanisms (decreasing the number of holes in nanocarbonic materials, proton conduction, and swelling of PVP), as well as their relative prevalence, determines the type of response.

### 5.3. Organic–Inorganic Nanohybrids Comprising CNHs Used as Sensing Layers in RH-Resistive Sensors

Due to the complementary and/or synergetic effects between inorganic and organic components, nanohybrid materials exhibit outstanding properties, such as good mechanical properties, biodegradability, tuned electrical properties, flexibility, enhanced surface area, porosity, and catalytic properties [94,95,96]. Therefore, nanohybrids have gained increased interest as a sensing layer in gas detection, with enhanced sensitivity, selectivity, and stability [97]. At the same time, recently, several CNHox-based nanohybrids were reported as sensing layers in resistive RH monitoring, as follows.

#### 5.3.1. Organic–Inorganic CNHox/KCl/PVP Nanohybrids Used as Sensing Layers in RH-Resistive Sensors

**Figure 19:**
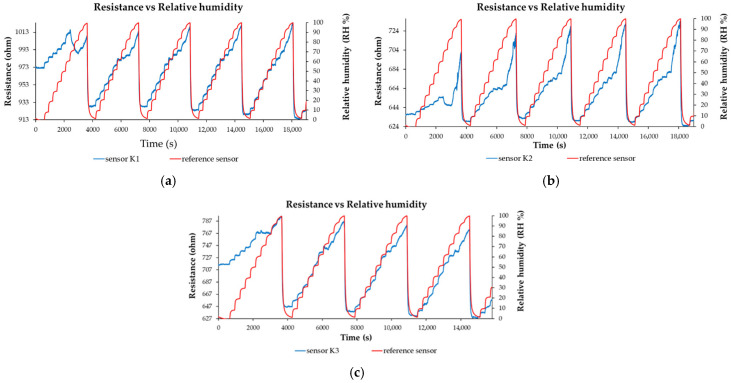
Resistance versus RH for the: (**a**) K1 sensor (CNHox/KCl/PVP, 7/1/2, *w*/*w*/*w*) and for the reference sensor; (**b**) K2 sensor (CNHox/KCl/PVP, 6.5/1.5/2, *w*/*w*/*w*) and for the reference sensor; and (**c**) K1 sensor (CNHox/KCl/PVP, 6/2/2, *w*/*w*/*w*) and for the reference sensor in several operating sequences [99].

Several nanohybrids based on CNHox/KCl/PVP, synthesized at mass ratios of 7/1/2, 6.5/1.5/2, and 6/2/2 (*w*/*w*/*w*), were used as sensing films in the design of the resistive RH sensor [98,99,100]. The associated RH sensors were abbreviated as K1, K2, and K3, respectively. The sensing structure comprised a Si substrate, a SiO_2_ layer, and an interdigitated transducer (IDT) based on Cr/Au electrodes. All RH detection measurements, presented in Figure 19a–c, were conducted by applying a current between the IDTs and measuring the voltage difference as the RH was varied from 0% to 100%. The resistance versus RH behavior of the manufactured sensors, based on CNHox/KCl/PVP as the sensing layer, was compared to that of a commercial capacitive RH sensor, used as a reference.

**Figure 20:**
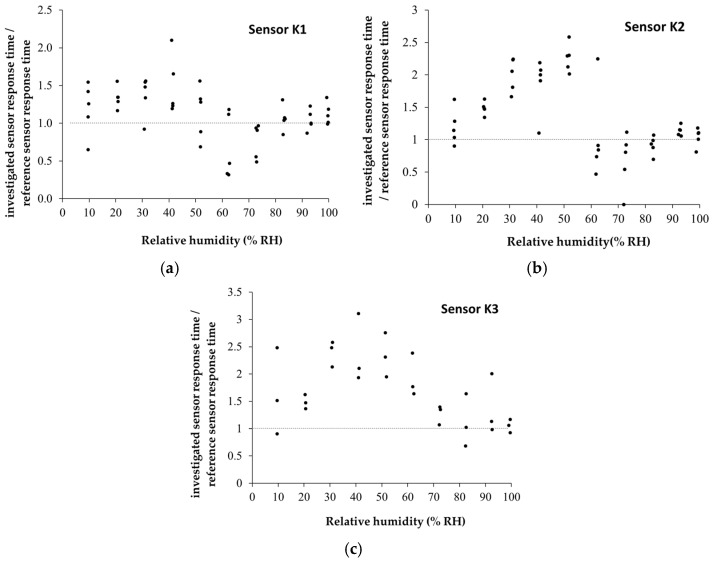
Graphical representations of the ratios between the response time of the manufactured CNHox/KCl/PVP-based sensors (**a**) K1 sensor; (**b**) K2 sensor; and (**c**) K3 sensor and the response time of the commercial sensor, measured in humid nitrogen, when varying RH from 0% to 100% [99].

The manufactured CNHox/KCl/PVP-based sensors showed room-temperature RH detection behavior comparable to that of the commercial capacitive RH sensor (Figure 19a–c). The devices are characterized by rapid response time (Figure 20), good sensitivity, and excellent linearity. For RH < 70%, the commercial sensor has a response time of 60 s ± 10 s, while for RH > 70%, the response time increases to approximately 90 s ± 10 s.

#### 5.3.2. Organic–Inorganic CNHox/TiO_2_/PVP Nanohybrids Used as Sensing Layers in RH-Resistive Sensors

**Figure 21:**
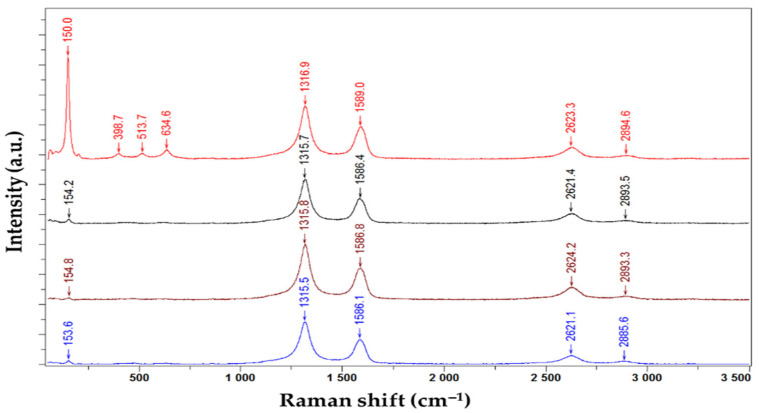
Raman spectra of the CNHox/TiO_2_/PVP nanocomposite solid-state film, with a 3:1:1 *w*/*w*/*w* mass ratio, deposited on glass, were recorded at four different points of the nanohybrid [100,101].

Serban et al. reported the use of CNHox/TiO_2_/PVP nanohybrid as a sensing layer for RH-resistive monitoring [101,102]. Three types of CNHox/TiO_2_/PVP nanohybrids were synthesized, at 1/1/1 (corresponding to the manufactured sensor T1), 2/1/1 (corresponding to the manufactured sensor T2), and 3/1/1/ (corresponding to the manufactured sensor T3) mass ratios (*w*/*w*/*w*). The synthesis of the nanohybrids was conducted in ethanol using an ultrasonic bath. The mutual interaction between CNHox, titania, and PVP was confirmed using Raman spectroscopy. The Raman spectra for the CNHox/TiO_2_/PVP at a 3:1:1 *w*/*w*/*w* mass ratio, deposited on the substrate, recorded at four points of the nanohybrid, are presented in Figure 21.

Three active Raman bands (D, G, and 2D) were measured at wavenumbers of 1316.9, 1589, and 2623.3 cm^−1^, which confirm the presence of CNHox [85,87]. Distinct anatase TiO_2_ bands, such as E_g_1 mode at 150 cm^−1^, B_1g_ at 398.7 cm^−1^, A_1g_ at 513,7 cm^−1^, and E_g_3 at 634.6 cm^−1^, were also recorded [103,104,105]. The corresponding peaks of PVP are likely hidden, most probably due to being masked by CNHox. The shift in Raman peak positions between individual TiO_2_ and CNHox and those of the same materials as components of the nanohybrid is a consequence of noncovalent interactions between them, such as hydrogen bonds and electrostatic interactions.

**Figure 22:**
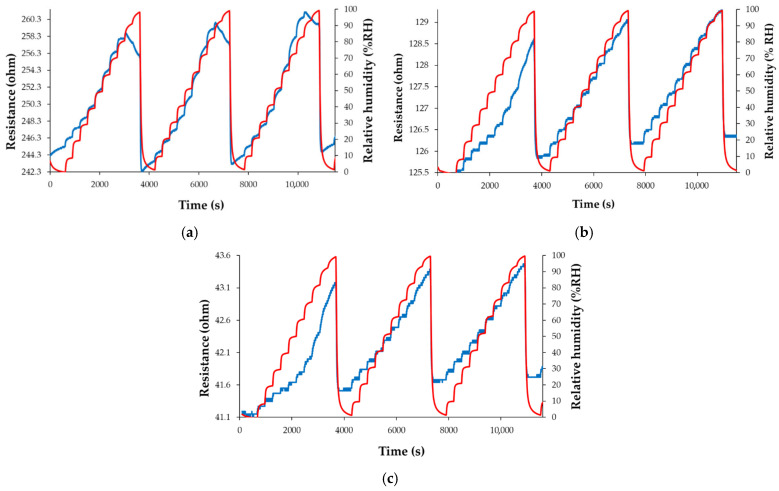
The response of the manufactured sensor: (**a**) T1; (**b**) T2; and (**c**) T3, as a function of time for three complete measurement cycles, when varying RH between 0% and 100%; “RH curve-red” shows the variation of the RH in the testing chamber, as indicated by the reference sensor [101,102].

The resistance of the CNHox-TiO_2_-PVP-based sensing film increases when RH increases from 0% to 80% RH (Figure 22a–c). For RH larger than 80%, subtle differences are recorded. Thus, the resistance of the T1 sensor moderately decreases with increasing RH, while the resistance of manufactured sensors T2 and T3 rises with RH. As shown in Figure 22a–c, the performance of the manufactured CNHox-TiO_2_-PVP-based RH sensors is comparable to that of a commercial RH sensor used as a reference.

#### 5.3.3. Organic–Inorganic CNHox/ZnO/PVP Nanohybrids Used as Sensing Layers in RH-Resistive Sensors

Serban et al. [106] deposited a ternary nanohybrid based on CNHox, ZnO, and PVP at a 5/2/1 mass ratio on a Si/SiO_2_ substrate using the drop-casting method. The morphology and composition of the sensing film were evaluated and confirmed through SEM and Raman spectroscopy. Experimental measurements showed that the resistance of the sensitive film increased with RH, varying from 0% to 100%. Increased RH sensitivity was recorded for RH > 60%. The response time of the manufactured CNHox-ZnO-PVP-based RH sensor was shown to be comparable to that of a commercially available capacitive RH sensor.

#### 5.3.4. Organic–Inorganic CNHox/SnO_2_/ZnO/PVP Nanohybrids Used as Sensing Layers in RH-Resistive Sensors

**Figure 23:**
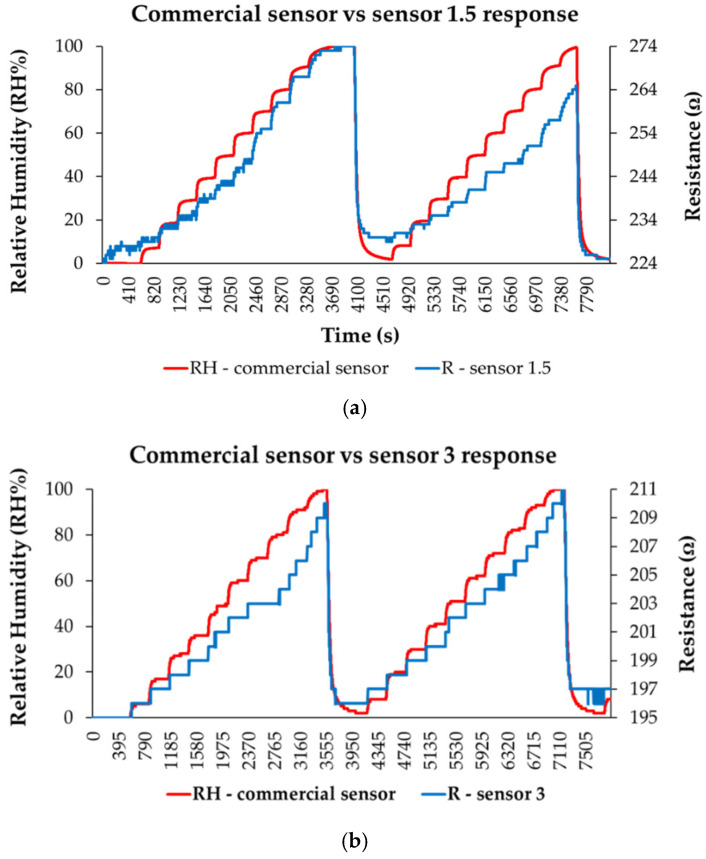
The response of: (**a**) “Sensor 1.5”, and (**b**) “Sensor “3” (“R curve”-blue) as a function of time for two measurement cycles, while increasing RH in 10 steps from 0% to 100% RH; “RH curve-red” shows the similar characteristic measured for a commercial, capacitive sensor [107].

A thin film based on a quaternary nanohybrid comprising CNHox/SnO_2_/ZnO/PVP was tested as a sensing layer in the resistive monitoring of RH [100]. Two CNHox/SnO_2_/ZnO/PVP-based sensing layers were synthesized and deposited, at a 1.5/1/1/1 (abbreviated as “sensor 1.5”) and 3/1/1/1 (abbreviated as “sensor 3”) mass ratio. The RH-sensing device consists of a Si/SiO_2_ substrate and interdigitated transducer (IDT) electrodes (Cr/Au). As depicted in Figure 23, for both CNHox/SnO_2_/ZnO/PVP nanohybrid-based sensing layers, the resistance increases when RH increases from 0% to 100%. Their experimental performance was compared to that of a commercial RH sensor, used as a reference.

**Figure 24:**
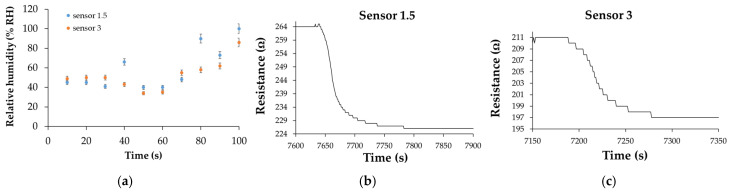
(**a**) Response times for “Sensor 1.5” and “Sensor 3” with RH increasing from 0% to 100%, with a 10% step, in the second measurement cycle recovery times for (**b**) “Sensor 1.5” and (**c**) “Sensor 3” after the second measurement cycle; the recovery time was measured by varying RH from 100% to 0% (clean, dry nitrogen) [107].

The response time of the CNHox/SnO_2_/ZnO/PVP-based manufactured RH sensors ranged from 35 to 100 s for both devices, as presented in Figure 24. The highest response times were recorded at RH > 70%, most likely due to a decrease in the number of active sites.

Figure 24b,c shows the recovery pattern for both quaternary nanohybrid-based RH-sensing layers measured when the RH from the testing box dropped from 100% to 0%. The calculated recovery times varied from 65 s to 100 s—values similar to those exhibited by the commercial sensor, which was employed as a reference.

#### 5.3.5. Organic–Inorganic CNHox/GO/SnO_2_/PVP Nanohybrid Used as Sensing Layers in RH-Resistive Sensors

**Figure 25:**
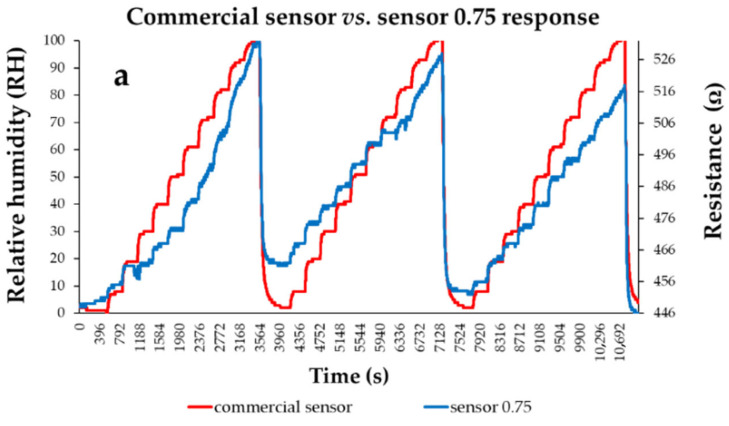

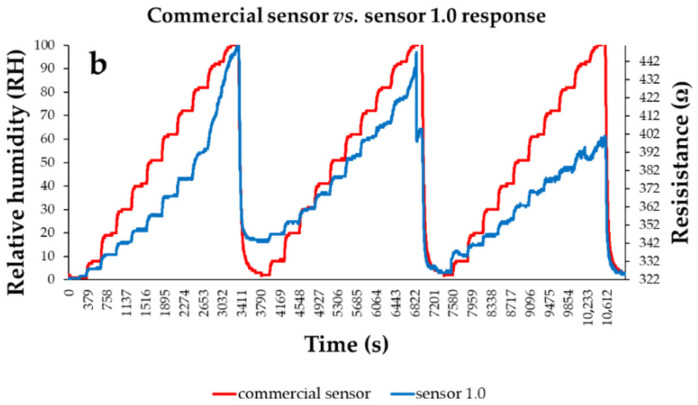
The response of (**a**) “Sensor 0.75” and (**b**) “Sensor 1.0” (“R curve-blue” curves) presented as a function of time for three measurement cycles while varying RH, in 10 steps, from 0% to 100%; “RH curve-red” shows the similar characteristic measured for a commercial, capacitive sensor [108].

Thin films based on a quaternary nanohybrid based on CNHox/GO/SnO_2_/PVP were also demonstrated as sensing layers in RH-resistive sensors [101]. Two CNHox/GO/SnO_2_/PVP-based sensing layers were synthesized, at 1/1/1/1 (abbreviated as “sensor 1”) and 0.75/0.75/1/1 (abbreviated as “sensor 0.75”) mass ratios. The RH-sensing device consisted of a Si/SiO_2_ substrate and interdigitated, Cr–Au transducer (IDT) electrodes. The composition and the morphology of the CNHox/GO/SnO_2_/PVP-based sensing films were explored and confirmed through X-ray diffraction (XRD), Scanning Electron Microscopy (SEM), and RAMAN spectroscopy. As depicted in Figure 25, for both CNHox/GO/SnO_2_/PVP nanohybrid-based RH-sensing layers employed, the resistance increased when varying RH from 0% to 100%. A notable characteristic of these two manufactured CNHox/GO/SnO_2_/PVP-based sensors is their low power consumption, which is below 2 mW. Their experimental performance was compared to that of a commercial capacitive RH sensor, used as a reference.

**Figure 26:**
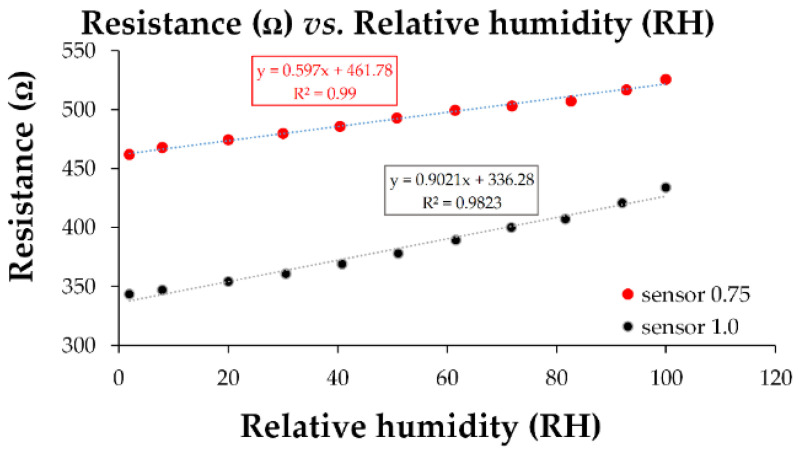
The transfer function of the quaternary CNHox/GO/SnO_2_/PVP nanohybrid-based resistive sensors in humid nitrogen (RH = 0–100%) [108].

The linearity of the manufactured CNHox/GO/SnO_2_/PVP-based sensors was excellent, as demonstrated by the transfer function shown in Figure 26.

Upon analyzing the comparative data presented in Table 2, it is evident that a sensing film based on CNHox combined with a hydrophilic polymer, such as PVP, demonstrates superior sensitivity compared to a sensitive layer solely based on CNHox. Furthermore, when CNH is used instead of CNHox, the resistance variation is observed to be higher than in all sensors utilizing CNHox. However, sensors that use CNH/PVP mixture exhibit instability at RH values exceeding 70%.

The sensitivity values reported for the sensing nanocomposites in Table 2 indicate that incorporating inorganic components into the mixture significantly enhances sensitivity. Notably, the addition of GO has a particularly beneficial impact on sensitivity toward water molecules.

In terms of response and recovery times, the data show that nanocomposites incorporating carbon nanohorns are strong candidates for designing commercial sensors, as their performance is comparable to that of existing commercial sensors. Another key observation is that the properties of the CNHox-based sensing layer can be effectively tuned by introducing various fillers into the nanocomposites. The choice of fillers, which influence the sensor properties, is guided by Pearson’s Hard–Soft Acid–Base (HSAB) theory, ensuring that the components of the sensing layer exhibit appropriate reactivity with water molecules.

Finally, it is essential to note that not all sensing layers are suitable for operation across the full 0–100% RH range, underscoring the need for careful selection and optimization of materials for specific applications.

The long-term stability and cyclic aging behavior of CNH-based sensors are crucial aspects to consider in the development of reliable humidity-sensing devices. The experimental findings indicate that all freshly prepared sensors exhibit an initial stabilization phase lasting 1–2 h, during which they adapt to the surrounding environment. After this initial period, the sensor responses become more reproducible. Regarding long-term stability, the use of CNHs as a key component of the sensing layer offers significant advantages. Carbon nanohorns are known for their stability in various environmental conditions, which contributes to the overall durability and reliability of the sensing device. When combined with hydrophilic polymers and other fillers, the sensing layer not only enhances the device’s response but also ensures consistent performance over extended periods of time. These characteristics make CNH-based sensors promising candidates for long-term humidity-sensing applications.

## 6. Sensing Mechanisms for RH-Resistive Monitoring Using CNHs and Their Nanocomposites/Nanohybrids

Any hypothesis regarding RH-resistive monitoring sensing mechanisms using CNHs (pristine and their derivatives) or nanocomposites/nanohybrids based on these nanocarbonic materials begins with the fact that CNHs are p-type semiconducting materials [111,112]. At the same time, it is to be expected that chemisorbed water molecules on the CNH surface operate as electron donors [113]. As the electron density increases, the positive charge concentration in CNHs decreases and, thus, p-type CNH-based films become more resistive. This scenario accounts for most of the reported experimental results concerning resistive RH sensors employing CNH-based sensing layers [82,83,89,90,91,92,93,98,99,100,101,102,103].

The interaction between water and CNHs (as p-type semiconducting materials) can also be discussed from the perspective of the Hard–Soft Acid–Base (HSAB) theory. This theory, proposed by Ralph Pearson in 1963, operates with Lewis acids and bases: a molecule capable of donating electron pairs acts as a base, while a molecule that acts as an electron acceptor is classified as an acid. Lewis acids and bases can be classified into three types: hard, soft, and borderline [114,115,116,117]. According to the HSAB concept, strong bases have a greater affinity for interacting with strong acids, while soft bases preferentially interact with strong acids. In contrast, borderline bases tend to interact with borderline acids. Given the above definitions and rules, due to the electron pairs in the oxygen atom, H_2_O is a typical example of a hard base and, consequently, has an affinity to interact with the positive charge carriers (hard acids) in the CNHs. In recent years, the HSAB theory has become a valuable tool for selecting sensitive materials for gas sensing, as well as for explaining specific reaction mechanisms [118,119,120].

At the same time, hydrogen bonds, as well as the electron-withdrawing effect of the carboxyl group (present in CNHox), influence the hole concentration in nanocarbonic films and can modulate RH sensitivity, ultimately affecting the RH sensor response [121].

Hysteresis behavior is crucial in evaluating the performance of RH sensors and their long-term stability, as it also provides an insight into the sensing mechanisms of water molecules. The hysteresis curves presented in Section 5, which represent the variation of both relative humidity and sensor resistance over time, reveal that freshly prepared sensors exhibit an initial “accommodation” phase with the environment. During the first one or two cycles of operation, the hysteresis curves show some deviation. However, after this initial stabilization period, the curves overlap consistently for all subsequent cycles, indicating improved reproducibility and stability of the sensor’s performance over time.

When comparing the CNH/PVP mixture with the CNHox/PVP mixture, the results demonstrate that CNHox exhibits superior hysteresis behavior. Specifically, CNHox exhibits a more consistent and predictable response as humidity increases from 0% to 100% and then decreases back to 0%. This improved behavior suggests that CNHox is a better candidate for humidity-sensing applications, particularly in scenarios requiring high accuracy and minimal hysteresis. These findings underscore the importance of material selection and composition in optimizing the performance of CNH-based RH sensors.

**Figure 27:**
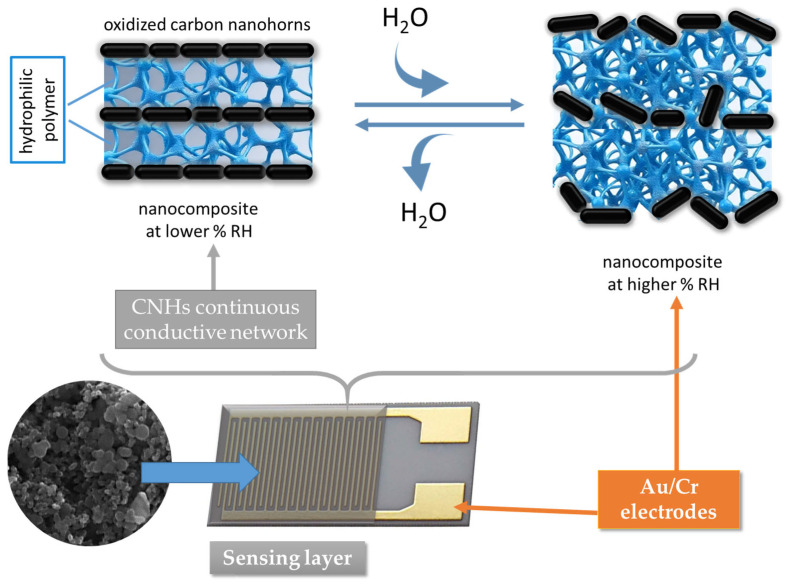
The swelling of the hydrophilic polymer included in CNH-based nanocomposites upon interaction with water.

The second plausible RH-sensing mechanism considered for the interpretation of the RH-sensing experimental results presented in the previous chapter is the swelling of the hydrophilic polymer employed in the nanohybrid sensing layer in contact with water [82,83,89,90,91,92,93,98,99,100,101,102,103]. PVP and PEG–PPG–PEG are dielectric polymers with hydrophilic properties, which swell upon interaction with moisture. The swelling of polymers leads to the displacement of the nanocarbonic particles, increasing the distance between the CNHs and decreasing the electrically percolating pathways, as depicted in Figure 27. Consequently, the sensing film resistance increases upon exposure to a higher level of RH because more water molecules move into the bulk of the nanocarbonic film.

PVP swelling is more pronounced at higher levels of RH. The swelling of PEG–PPG–PEG is somewhat different from that of PVP and requires a supplementary explanation. PEG–PPG–PEG is less hydrophilic than PVP, and more water molecules are needed to initiate swelling. For RH < 40%, the resistance of PEG–PPG–PEG-based films exhibits a relatively low increase with RH, while for RH > 65% the sensing layer resistance increases sharply with RH (switch-type behavior).

A third plausible RH-sensing mechanism that needs to be discussed is based on the fact that, given the type of employed analyte (water), protonic conduction must be considered as a potential contributor to RH sensing. This type of sensing mechanism refers to the dissociation of water (a major contributor) and/or the ionization of carboxylic acids (from CNHox). The adsorbed water molecules on the surface of CNHox may dissociate into H^+^ and OH^−^. The protons generated by water dissociation and the ionization of carboxylic groups decrease the overall electrical resistance of the sensing layer [122]. However, since the experimental data presented in the previous section show an increase in resistance with RH, one can conclude that the contribution of this third RH-sensing mechanism, based on proton tunneling, is relatively small compared to the impact of the first two sensing mechanisms discussed above.

Beyond the three RH-sensing mechanisms, the individual contributions of other components in the CNH-based sensing films, as well as the mutual interactions between them, may also play a crucial role in the overall RH-sensing mechanism.

The selectivity of CNH-based sensors also needs to be taken into consideration. The presented data focus primarily on nitrogen with varying humidity levels, as the primary goal was to evaluate the sensors’ performance in detecting water vapor. However, there is evidence in the literature that CNHs and their oxidized derivatives (CNHox) can be utilized for detecting other gases. For instance, Keshtkar et al. demonstrated that a mixture of CNHs with tin oxide could effectively detect gaseous carbohydrates, highlighting the potential of CNH-based materials for broader gas-sensing applications [123].

The findings suggest that the structure of the filler, along with the composition of the polymer and CNH or CNHox, can significantly influence the sensitivity and selectivity of the sensor. By carefully tuning these parameters, it is possible to enhance the sensor’s ability to discriminate between water vapor and other gases, thereby improving its selectivity. Future work could expand on this by systematically testing CNH-based sensors with a wider range of gases to further validate their selectivity and versatility.

The performance of sensors at non-room temperatures is an important factor for real-world applications. However, there is currently a lack of extensive data regarding the behavior of CNH-based sensing layers under varying temperature conditions. As a consequence, this review focused on the use of CNHs and CNH-based nanohybrids in resistive humidity sensors at room temperature. While this provides valuable insights into their potential for humidity sensing, further studies are required to evaluate their performance across a broader temperature range. In the literature, very few studies address the temperature-dependent sensing behavior of CNH-based materials. For instance, Keshtkar et al. (2017) investigated tin dioxide quantum dots/carbon nanohorns nanohybrids as low-temperature natural gas sensors [123]. While this study highlights the feasibility of CNH-based materials for gas sensing at non-room temperatures, it does not involve the use of hydrophilic polymers, which are a key component of the systems discussed in this review.

Future work should focus on systematically studying the temperature dependence of CNH-based humidity sensors, particularly for devices incorporating hydrophilic polymers. Understanding how temperature affects the interaction between water molecules and the sensing layer, as well as the overall sensor performance, will be critical for expanding the applicability of these materials in diverse environmental conditions

## 7. Why Are CNHs Used Less Frequently than CNTs and Graphene Derivatives for Resistive RH Sensing? Possible Opportunities and Future Research Directions

CNHs with hydrophilic properties (CNHox or holey CHNs) seem to be viable solutions as a sensing layer within resistive RH sensors design due to specific outstanding properties:

- *The absence of metallic particles as impurities*—Synthesis of the CNHs is conducted in the absence of metal catalyst [60].
- *High Surface Area*—CNHox possesses a large specific surface area (1300–1400 m^2^/g BET), providing more sites for the adsorption of water, which increases its RH sensitivity [85].
- *Tunable Surface Chemistry*—The versatile hydrophilization of CNHs (through oxidation in solution or plasma treatment) allows for the optimization of the response of the sensor to different RH levels [60].
- *Good Electrical Conductivity*—CNHox retains good electrical conductivity even after hydrophilization, which is a key feature for resistive RH sensors. The interaction of water molecules with the CNHox surface can modify the electrical resistance of the sensing film, allowing for accurate RH monitoring [69].
- *RT Operation*—CNHox-based sensors can operate at RT, which is an advantage for manufacturing ultralow-power resistive sensors [99,100,101].
- *Excellent Linearity and Stability*—CNHox-based RH sensors have demonstrated excellent linearity in their response across a wide range of RH levels, and have also shown good stability over time [89].
- *Rapid Response and Recovery Times*—The large surface area and tunable surface chemistry of CNHox contribute to obtaining a fast response and recovery time [90].
- *Compatibility with other materials*—CNHox can be easily incorporated into nanocomposites or nanohybrids with different materials, like polymers (e.g., PVP, PEG–PPG–PEG), carbonic materials (GO), and metal oxides (e.g., TiO_2_, ZnO, SnO_2_), further enhancing their sensing properties and enabling the development of RH sensors [57].
- *Potential for Flexible and Wearable Sensors*—The ability to create thin films and dispersions makes CNHox suitable for integration into flexible and wearable sensors, expanding the possible applications of RH sensors [60].

Despite their exceptional properties, carbon nanohorns (CNHs) are used less frequently than other nanocarbon materials, such as carbon nanotubes (CNTs), graphene oxide (GO), and reduced graphene oxide (rGO), in RH monitoring. CNHs hold immense potential in sensing devices, particularly in resistive RH sensors, due to their unique characteristics. However, their adoption remains limited compared to other nanocarbon materials. This limitation can be attributed to several challenges, including the tendency of CNHs to aggregate during synthesis, the lack of advanced predictive modeling, and an incomplete understanding of their properties.

This issue is not restricted to RH sensors but also extends to gas sensors in general. The following question arises: why are CNHs not as widely utilized as other carbon allotropes? Below, several responses to this question are outlined and future research directions are proposed that could enhance the adoption and utility of CNHs in sensing applications.

### Future Directions

- Overcoming aggregation of carbon nanomaterials. CNHs tend to aggregate into spherical clusters, making it difficult to disperse them individually and create a homogeneous sensing layer. For many years, this was the major limitation in the chemistry of CNHs. Recently, this drawback was overcome by employing a new technique for separating the clusters into distinct nanohorns [60]. Further refinement of these methods is necessary to leverage the potential of CNHs fully.
- Another possible limitation is that the low symmetry of CNHs reduces the accuracy of predictive simulations. For these reasons, CNHs are less understood and used than other nanocarbonic materials [60]. Thus, several refined computational techniques, such as molecular dynamics (MD) and Monte Carlo (MC) simulations, as well as density functional theory (DFT) calculations, can offer more opportunities for achieving an optimal level of hydrophilicity or proper functionalization.
- Functionalization of CNHs is another way to improve the properties of the sensing layers. The tunable surface chemistry of CNHs offers a unique opportunity to optimize their sensing performance. Functionalization with polymers, metal oxides, or other nanomaterials can significantly enhance their sensitivity and selectivity. Recent advancements in surface functionalization strategies have opened new possibilities for enhancing the performance of CNHs in sensing devices. Among these, the use of bio-linkers has emerged as a promising approach, particularly for applications requiring high sensitivity and specificity, such as biosensing and environmental monitoring. Bio-linkers, including proteins, peptides, and other biomolecules, can be covalently or non-covalently attached to the surface of CNHs, enabling selective interactions with target analytes. This functionalization not only enhances the selectivity of CNH-based sensors but also facilitates the detection of biological molecules, including enzymes, antibodies, and DNA.
- For example, bio-linkers can be utilized to create CNH-based sensors for detecting specific gases or humidity levels in environments where biological interactions are significant. Additionally, bio-functionalized CNHs can be integrated into wearable or portable sensing platforms, paving the way for real-time monitoring of physiological parameters. Another advantage of bio-linkers is their ability to minimize non-specific interactions, which is crucial for improving the accuracy and reliability of the sensing device.
- Incorporating these recent strategies into the design of sensing layers would provide a more comprehensive perspective on the potential of CNHs in sensing applications. Future research should focus on optimizing the stability and compatibility of bio-functionalized CNHs to ensure their effectiveness in practical applications [124].
- Expanding the use of CNHs in flexible and wearable sensors is possible due to their compatibility with flexible substrates, which positions them as ideal candidates for next-generation wearable sensors, enabling real-time and portable monitoring.
- Developing scalable and cost-effective synthesis methods will ensure the wider adoption of CNHs, making them a sustainable alternative to metal-based sensing materials. A critical factor influencing the adoption of CNHs in sensing applications is their cost of synthesis compared to other nanocarbon materials, such as carbon nanotubes (CNTs) and graphene. While CNHs benefit from metal-free synthesis, which eliminates the need for expensive metal catalysts, their production processes, such as laser ablation or arc discharge, tend to be energy-intensive and require specialized equipment. This can increase the overall cost, particularly when scaling up production. In contrast, CNTs, especially multi-walled carbon nanotubes (MWCNTs), are often synthesized using chemical vapor deposition (CVD), a well-established and scalable method that, although reliant on metal catalysts, makes CNTs more cost-competitive in large-scale production. Similarly, graphene derivatives such as graphene oxide (GO) and reduced graphene oxide (rGO) are typically produced through the chemical exfoliation of graphite, which is a relatively low-cost and scalable process due to the abundance of graphite as a raw material. However, the additional purification steps required to remove metallic impurities from CNTs and the complex reduction processes for GO and rGO can offset some of their cost advantages. CNHs, on the other hand, offer unique performance benefits, such as high purity and the absence of metallic impurities, directly from synthesis, which can reduce post-processing costs. To make CNHs more economically viable, further research is needed to develop energy-efficient synthesis methods, such as plasma-based or solution-phase processes, which could lower production costs while maintaining their high-quality properties. Addressing these cost disparities will be crucial for positioning CNHs as a sustainable and cost-effective alternative to CNTs and graphene in sensing applications [125].

By addressing these challenges and leveraging their unique properties, CNHs have the potential to revolutionize the field of sensing technology. Researchers are encouraged to explore these future directions, which could lead to significant advancements in RH sensing, gas detection, biomedical devices, and environmental monitoring.

We believe that carbon nanohorns hold significant future potential and will be utilized in an increasing number of applications, particularly in gas sensing, biomedicine, and energy storage.

## 8. Conclusions

This paper reviews and analyzes recent advancements in resistive humidity sensors based on CNHs, with a focus on their operation above the percolation threshold within a dielectric matrix. The initial sections of the review discuss various methods for synthesizing CNHs, strategies for their hydrophilization, and their key physical, chemical, and electronic properties. Subsequent sections delve into the design of RH-resistive sensors utilizing CNH-based materials as sensing films, as well as the synthesis and performance of several CNH-based sensing materials for resistive RH monitoring. These include pristine and functionalized CNHs, nanocomposites with hydrophilic polymers (e.g., PVP, PEG–PPG–PEG), other nanocarbon materials (e.g., graphene oxide), and nanohybrids incorporating CNHs with metal oxide semiconductors (e.g., SnO_2_, TiO_2_, ZnO) or hygroscopic inorganic salts (e.g., KCl). These materials are compared in terms of their linearity, sensitivity, response time, and recovery time.

The stability and cyclic aging behavior of CNH-based sensors are critical for developing reliable humidity-sensing devices. In this study, the materials tested represent an innovative approach, with experiments conducted over multiple operating cycles (up to five cycles) and measurements taken at one-second intervals. The findings revealed that freshly prepared sensors undergo an initial stabilization phase of 1–2 h, during which they adapt to their environment, after which their responses become more consistent. Regarding long-term stability, CNHs offer significant advantages due to their robustness under various environmental conditions, which enhances the durability and reliability of the sensing devices. When combined with hydrophilic polymers and other fillers, the sensing layers not only improve response performance but also ensure consistent functionality over extended periods. These properties make CNH-based sensors promising candidates for long-term humidity-sensing applications.

The fifth section of the review outlines several sensing mechanisms that explain RH detection using CNH-based sensing layers in resistive structures. The sixth section highlights the key properties of CNHs that make them suitable for resistive RH detection. At the same time, it also discusses why CNHs, compared to other carbon nanomaterials like graphene, graphene oxide (GO), reduced graphene oxide (rGO), single-walled carbon nanotubes (SWCNTs), and multi-walled carbon nanotubes (MWCNTs), are currently less widely used for resistive RH detection.

Despite these challenges, CNHs demonstrate immense potential for future applications, particularly in gas sensing, biomedicine (e.g., drug delivery), and energy storage (e.g., supercapacitors and batteries).

To fully realize the potential of CNHs in sensing applications, several challenges must be addressed. Overcoming the aggregation of carbon nanomaterials and improving the functionalization of CNHs are critical steps for enhancing the properties of sensing layers. The tunable surface chemistry of CNHs provides a unique opportunity to optimize their sensing performance. Functionalization with polymers, metal oxides, or other nanomaterials can significantly improve sensitivity and selectivity. Additionally, the compatibility of CNHs with flexible substrates opens up opportunities for their use in flexible and wearable sensors. Developing scalable and cost-effective synthesis methods is essential for the broader adoption of CNHs, positioning them as a sustainable alternative to metal-based sensing materials.

## Figures and Tables

**Table 1:** Examples of nanocarbon-based sensing layers used as sensing elements in the design of RH-resistive sensors.

Type of Nanocarbonic Material	Substrate	Measured RH Range (%)	Reference
Graphene	Si/SiO_2_	1–96	[31]
Graphene/ZnO	Glass	15–86	[32]
Graphene/Poly(3,4-ethylenedioxythiophene)-polystyrene sulfonate	Si/SiO_2_, Kapton, PET, Paper	30–95	[33]
Graphene oxide (GO)	Si/SiO_2_	40–88	[34]
Reduced graphene oxide/Poly (diallyldimethylammonium chloride) (PDAC)	Glass	20–70	[35]
Multi-walled carbon nanotubes (MWCNTs)	Polyimide	10–90	[36]
MWCNTs/Polyvinylpyrrolidone (PVP)	Quartz	11–94	[37]
Pristine carbon nano-onions (CNOs)/PVP at 1/1 *w*/*w* ratio	Polyimide	0–100	[38]
Pristine CNOs/Polyvinyl Alcohol (PVA) at a 1/1 and 2/1 *w*/*w* ratios	Si/SiO_2_	5–95	[39]
MWCNTs/Polyimide	Si_3_N_4_	10–95	[40]
Hydrogenated amorphous carbon	Synthetic resin FR2	10–100	[41]
Carbon-black/PVA	glass	10.9–73.7	[42]
Shellac-derived carbon (SDC)	Si/SiO_2_	10–90	[43]
Carbon nano coils (CNCs)	Liquid crystal polymer (LCP)	4–95	[44]
Porous carbon nanofiber	Cellulose	13–97.3	[45]
Carbon nanosheets and nanohoneycombs	Si (100)	11–95	[46]
Carbon quantum dots	Glass sheet	7–95	[47]
Pyrolyzed bamboo	α-alumina	0–96	[48]
Biochar	α-alumina	5–100	[49]
Graphene quantum dots	Si/SiO_2_	15–80	[50]
Multi-walled carbon nanotubes (SWCNT)/Pt/P_2_O_5_	Ceramic	1–90	[51]
SWCNTs/PVA filaments	Textile cloth	60–100	[52]
Chitosan/ZnO/SWCNTs	Polyimide	11–97	[53]
Carbon nanodots	Polytetrafluoroethylene (PTFE)	11–94	[54]
Carbon ink	PET flexible substrate	2–95	[55]

**Table 2:** Comparison of sensitivity for resistive RH monitoring for sensing layers based on CNHox and their nanocomposites, and two commercially available sensors.

Sensing Layer Composition (*w*/*w*)	Sensor Type	Sensitivity ΔR/ΔRH (Ω/%RH)	Response Time (s)	Recovery Time (s) (RH 100% to 0%)	Linearity Domain (%RH)	Operating Range (%RH)	Ref.
CNHox	resistive	0.017 ± 0.004	7–9	N/A	10–90	10–90	[87]
CNH/PVP	resistive	1.680 ± 0.060	4–6	50–54	0–70%	0–70%	[92,93]
CNHox/PVP 2/1	resistive	0.025 ± 0.005	4–6	90–95	0–40	0–100	[89]
		0.058 ± 0.010	4–6		40–100		
CNHox/PVP 1/1	resistive	0.020 ± 0.005	4–6	90–95	0–100	0–100	
CNHox/PEG–PPG–PEG 1/6	resistive	N/A	N/A	N/A	0–60	0–100	
					60–100		
GO/CNHox/PVP 1/3/1	resistive	0.047 ± 0.003	4–8	85–90	0–100	0–100	[90,91]
GO/CNHox/PVP 1/2/1	resistive	0.067 ± 0.004	4–8	85–90	0–100	0–100	
GO/CNHox/PVP 1/1/1	resistive	0.170 ± 0.020	4–5	100–105	0–100	0–100	
CNHox/KCl/PVP 7/1/2	resistive	0.917 ± 0.320	4–11	55–60	0–60	0–60	[99]
CNHox/KCl/PVP 6.5/1.5/2	resistive	0.626 ± 0.280	4–14	55–60	0–60	0–60	
CNHox/KCl/PVP 6/2/2	resistive	1.398 ± 0.450	5–15	55–60	0–60	0–60	
CNHox/TiO_2_/PVP 1/1/1	resistive	0.189 ± 0.450	6–12	N/A	0–80	0–80	[101,102]
CNHox/TiO_2_/PVP 2/1/1	resistive	0.030 ± 0.010	3–15	43–45	0–100	0–100	
CNHox/TiO_2_/PVP 3/1/1	resistive	0.018 ± 0.010	3–15	43–45	0–100	0–100	
CNHox/ZnO/PVP 5/2/1	resistive	0.013 ± 0.050	7–12	85–90	0–60	0–100	[106]
		0.037 ± 0.089			60–100		
CNHox/SnO_2_/ZnO/PVP 1.5/1/1/1	resistive	0.400	4–8	62–65	0–70	0–70	[107]
CNHox/SnO_2_/ZnO/PVP 3/1/1/1	resistive	0.128	4–10	62–65	0–100	0–100	
CNHox/GO/SnO_2_/PVP 0.75/0.75/1/1	resistive	0.902	4–8	115–120	0–80	0–80	[108]
CNHox/GO/SnO_2_/PVP 1/1/1/1	resistive	0.597	4–8	160–165	0–80	0–80	
Texas Instruments HDC3120	capacitive	N/A	4	100	0–100	0–100	[109]
Sensirion SHT4/A-AWSB	capacitive	N/A	4	100	0–100	0–100	[110]

## Data Availability

Not applicable.

## Abbreviations

The following abbreviations are used in this manuscript:

CNHs	Carbon nanohorns
CNHs-F	Fluorinated carbon nanohorns
CNHox	Oxidized carbon nanohorns
CNHox-F	Oxi fluorinated carbon nanohorns
CNCs	Carbon nano coils
CNCs	Carbon nano tubes
GO	Graphene oxide
IDT	Interdigitated
LCP	Liquid crystal polymer
PDAC	Poly(diallyldimethylammonium chloride)
PEG	Poly(ethylene glycol)
PEG–PPG–PEG	Poly(ethylene glycol)-block-poly(propylene glycol)-block-poly(ethylene glycol)
PET	Polyethylene terephthalate
PPG	propylene glycol
PVA	Polyvinyl Alcohol
PVP	Polyvinylpyrrolidone
PTFE	Polytetrafluoroethylene
MWCNTs	Multi-walled carbon nanotubes
RH	Relative humidity
SWCNTs	Single-walled carbon nanotubes
SWNHs	Single-walled nanohorns
SDC	Shellac-derived carbon
